# Beneficial Effects of Exogenous Melatonin and Dopamine on Low Nitrate Stress in *Malus hupehensis*

**DOI:** 10.3389/fpls.2021.807472

**Published:** 2022-01-28

**Authors:** Peihua Du, Baoying Yin, Yang Cao, Ruoxuan Han, Jiahao Ji, Xiaolong He, Bowen Liang, Jizhong Xu

**Affiliations:** College of Horticulture, Hebei Agricultural University, Baoding, China

**Keywords:** nitrate deficiency, melatonin, dopamine, nitrate uptake, *Malus hupehensis*

## Abstract

Malus hupehensis, as an apple rootstock, is an economically important tree species popular due to its excellent fruit yield and stress resistance. Nitrogen is one of the critical limiting factors of plant growth and fruit yield, so it is crucial to explore new methods to improve nitrogen use efficiency. Melatonin and dopamine, as multifunctional metabolites, play numerous physiological roles in plants. We analyzed the effects of exogenous melatonin and dopamine treatments on the growth, root system architecture, nitrogen absorption, and metabolism of *M. hupehensis* when seedlings were exposed to nitrate-deficient conditions. Under low nitrate stress, plant growth slowed, and chlorophyll contents and ^15^NO_3_^–^ accumulation decreased significantly. However, the application of 0.1 μmol/L melatonin or 100 μmol/L exogenous dopamine significantly reduced the inhibition attributable to low nitrate levels during the ensuing period of stress treatment, and the effect of dopamine was more obvious. In addition to modifying the root system architecture of nitrate-deficient plants, exogenous melatonin and dopamine also changed the uptake, transport, and distribution of ^15^NO_3_^–^. Furthermore, both exogenous melatonin and dopamine enhanced tolerance to low nitrate stress by maintaining the activity of enzymes (NR, NiR, GS, Fd-GOGAT, and NADH-GOGAT) and the transcription levels of related genes involved in leaf and root nitrogen metabolism. We also found that exogenous melatonin and dopamine promoted the expression of nitrate transporter genes (*NRT1.1*, *NRT2.4*, *NRT2.5*, and *NRT2.7*) in nitrate-deficient plant leaves and roots. Our results suggest that both exogenous melatonin and dopamine can mitigate low nitrate stress by changing the root system architecture, promoting the absorption of nitrate, and regulating the expression of genes related to nitrogen transport and metabolism. However, according to a comprehensive analysis of the results, exogenous dopamine plays a more significant role than melatonin in improving plant nitrogen use efficiency.

## Introduction

Nitrogen (N) is required in large amounts by plants, and plant growth and productivity can be restricted when it is deficient (Maathuis, 2009; Liu et al., 2021). However, soils frequently fail to provide sufficient levels of N to sustain optimal plant growth and development (Jia et al., 2019). The application of N fertilizer has contributed greatly to crop yields, largely alleviating the pressure caused by the global population surge. Nevertheless, there is evidence that crops use only 30–50% of the N applied, and the loss of reactive N from agricultural soils causes serious environment pollution (Guo et al., 2010; Su et al., 2020). Thus, it is necessary to enhance crop nitrogen use efficiency (NUE) to improve yield quality with low N investment (Liu et al., 2021).

Nitrate (NO_3_^–^) is one of the two major forms of inorganic N absorbed from the soil by plants. Under aerobic conditions, NO_3_^–^ is the predominant form of N (Wang Y. Y. et al., 2018). NO_3_^–^ is principally absorbed into roots through NO_3_^–^ transporters from the nitrate transporter (NRT) families (Kiba et al., 2012; Lezhneva et al., 2014). During the assimilation process for conversion into amino acids, NO_3_^–^ is first reduced and transformed into nitrite (NO_2_^–^) by nitrate reductase (NR) in the cell cytosol and then reduced and transformed into ammonium (NH_4_^+^) by nitrite reductase (NiR) in chloroplasts or plastids (Xu et al., 2012). Subsequently, NH_4_^+^ is assimilated to glutamine and glutamate by glutamine synthetase (GS) and glutamate synthase (GOGAT), respectively (Huang et al., 2018). NO_3_^–^ can be stored in large quantities in plant vacuoles and recovered as needed; however, the NO_3_^–^ content available from soil fluctuates sharply over short distances and periods (Jia et al., 2019). Therefore, to determine the ratio among assimilation, tissue distribution, and vacuolar storage after NO_3_^–^ absorption by plants from roots to optimize access to NO_3_^–^ under limiting conditions, plants must first establish mechanisms to monitor external NO_3_^–^ supply levels efficiently, then respond to N demand from the various plant tissues, and eventually systematically transmit this information to activate metabolic enzymes and transporters via transcription or posttranscriptional regulation (Wang Y. Y. et al., 2018).

Melatonin (*N*-acetyl-5-methoxytryptamine) is a pleiotropic molecule with many diverse functions in plants (Arnao and Hernandez-Ruiz, 2019). After it was first identified in vascular plants (Dubbels et al., 1995; Hattori et al., 1995), numerous studies revealed that it is a general antioxidant compound capable of interacting with huge amounts of reactive oxygen species (ROS) and reactive nitrogen species (RNS), making it a significant factor in the stability of biological membranes, particularly mitochondrial membranes (Wang et al., 2017). Plant melatonin not only takes on an antioxidative role to remove ROS, but also exerts substantial effects in many physiological processes, such as seed germination, growth, flowering, and protection against biotic and/or abiotic stressors (Arnao and Hernandez-Ruiz, 2019; Liang et al., 2019). Recently, the identification of the first plant melatonin receptor in *Arabidopsis thaliana* led to consideration of this regulatory molecule as a new plant hormone (Wei et al., 2018).

Dopamine (3-hydroxytyramine or 3,4-dihydroxyphenethylamine) is a catecholamine, which are widespread in both animals and plants. It is a neurotransmitter in mammals that has been implicated in processes such as reward, addiction, and hormonal secretion (Wang S. et al., 2018). Since dopamine was first discovered in plants (Kimura, 1968), several studies have uncovered its important role in plants. However, in contrast to the huge amount of knowledge about its functions in mammals, the physiological significance of dopamine in plants remains unclear. Tyramine and L-dopa, the precursors of dopamine, are generated via either tyrosine decarboxylase (TYDC) or tyrosine hydroxylase (TH) (Kulma and Szopa, 2006; Rezaei et al., 2016), and the biosynthetic pathways of dopamine in plants resemble those in mammals (Liu Q. W. et al., 2020). However, dopamine biosynthesis pathways differ among plants. Dopamine is a water-soluble molecule with stronger antioxidant activity in plants than glutathione, similar to gallocatechin gallate and ascorbic acid (Kulma and Szopa, 2006). Dopamine also plays a critical role in the response of plants to abiotic stress, and can promote the growth of plants under various stressful environmental conditions (Liu Q. W. et al., 2020). More recent researches have reported that dopamine can enhance plant tolerance of drought, salt stress, and nutrient deficiency (Li et al., 2015; Liang et al., 2017, 2018a). In addition, exogenous dopamine was shown to increase the uptake of nutrients under drought stress conditions (Liang et al., 2018a). Recent studies have shown that DOH-CB protein can bind to dopamine through the induction of auxin (Verelst and Asard, 2004). Therefore, DOH-CB protein may be the receptor of plant catecholamines, and the binding between them may be induced by auxin (Liu Q. W. et al., 2020).

Over the past decade, significant progress has been made in understanding how NO_3_^–^ absorption, transport, and assimilation are modulated (Kiba et al., 2012; Lezhneva et al., 2014; Wang Y. Y. et al., 2018), and how exogenous substances such as plant hormones play a role under low N conditions (Su et al., 2020). However, the effects of melatonin and dopamine on the transcriptional regulation of genes related to NO_3_^–^ uptake and metabolism under low-NO_3_^–^ stress have not been studied systematically to our knowledge. ^15^N labeling stable isotope technique can provide important insights into N uptake by roots from soil profiles (Shiva et al., 2016), and the effects of melatonin on nutrient absorption under drought conditions have been studied using ^15^N tracer (Liang et al., 2018b). Here, the research also described applied such tracers to study the effects of melatonin and dopamine on N uptake under low NO_3_^–^ conditions.

Numerous studies have shown that melatonin and dopamine are involved in the response to various environmental stresses in plants, including nutrient deficiency (Li et al., 2016; Liu X. M. et al., 2020). To date, most studies have focused on enhancing the chlorophyll level, photosynthetic performance, and antioxidant enzyme activity associated with abiotic stress, less attention has been paid to nutrient utilization efficiency under low-NO_3_^–^ stress. Here, a hydroponics system was used to test whether the application of exogenous melatonin and dopamine has a positive effect on *M. hupehensis* under low-NO_3_^–^ stress. We investigated photosynthesis, root system structure, ^15^NO_3_^–^ absorption and distribution, and related enzyme activity and gene expression with the aim to clarify the regulatory mechanisms of melatonin and dopamine on nutrient uptake and utilization under low-NO_3_^–^ stress.

## Materials and Methods

### Plant Materials and Growing Conditions

The research was conducted at Hebei Agricultural University, Baoding (38°23′ N, 115°28′ E), Hebei, China. Triploid and apomixis-type *M. hupehensis* seeds were collected from Pingyi (35°07′ N, 117°25′ E), Shandong, China. For seed germination, cold stratification was required for 50 days at 4°C. After germination, three seeds were planted in each plastic pot filled with sand, and placed in a greenhouse under natural light and temperature conditions. The design of our hydroponic system was consistent with that of Liang et al. (2017). After 40 days of growth, we selected plants of similar size (8–9 leaves, about 8 cm high) and transferred them to plastic tubs wrapped with black plastic to block light exposure to the root systems containing 10 L of Hoagland semi-strength nutrient solution. We maintained the oxygen content in the nutrient solution by installing air pumps (Bai et al., 2010). The pH of the nutrient solution was adjusted to 6.5 ± 0.1 with H_3_PO_4_, and the solution was refreshed every 5 days.

### Experimental Design

The melatonin and dopamine concentrations in our experiments were consistent with those described in our previous studies (Li et al., 2016; Liang et al., 2017, respectively). After 10 days of preconditioning, *M. hupehensis* seedlings supplied K^15^NO_3_ as the only source of N were divided into six experimental groups: control: 2 mM K^15^NO_3_ solution (CK); NO_3_^–^-deficient treatment: 0.1 mM K^15^NO_3_ solution (ST); melatonin control: 2 mM K^15^NO_3_ solution with 0.1 μmol/L melatonin (MCK); melatonin- and NO_3_^–^-deficient treatment: 0.1 mM K^15^NO_3_ solution with 0.1 μmol/L melatonin (MST); dopamine control: 2 mM K^15^NO_3_ solution with 100 μmol/L dopamine (DCK); and dopamine- and NO_3_^–^-deficient treatment: 0.1 mM K^15^NO_3_ solution with 100 μmol/L dopamine (DST). The K^15^NO_3_ was produced by the Shanghai Research Institute of Chemical Industry (abundance of 98.14%). Each of the six groups included three replicates with 150 seedlings, and the trial spanned 20 days. Plant growth measurements were made on Days 0 and 20, while root architecture and enzyme activity were analyzed on Day 20. Chlorophyll concentrations and gene expression were determined on Days 0, 5, 10, 15, and 20. Fine roots and mature leaves were collected for different measurements, which included enzyme activity and gene expression.

### Growth Measurements

The plant length (PL) and trunk diameter (TD) was measured after 20 days of treatment. The whole plant harvested from each treatment was divided into root, stem, and leaf sections. Then, each sample was fixed at 105°C for 15 min and oven-dried at 70°C for at least 72 h to determine the dry weight. The total dry weight (TDW) was defined as the sum of the dry mass of roots, stems, and leaves. The root-to-stem ratio (RSR) was calculated by dividing the root dry weight by the stem dry weight. The relative growth rate (RGR) was calculated by the equation of Liang et al. (2018a). After being individually ground and sieved, fully triturated samples of leaf, stem and root were used for determination of ^15^N.

### Chlorophyll Extraction and Measurement

The leaves in the middle of the plant were extracted with 80% acetone for more than 24 h, and the pigment content was determined by spectrophotometry, as described by Arnon (1949).

### Investigation of Root Architecture

The root systems were cleared of impurities, without damaging the systems, with tap water, distilled water, and double-steamed water. We used a scanner to image the root system and analyzed the root architecture via the WinRHIZO^®^ image analysis system (V4.1c; Régent Instruments, QC, Canada).

### Determination of ^15^N Uptake Flux and Partitioning

Nitrogen stable isotope ratio was determined using an elemental analyzer (Flash EA 1112HT, Thermo Fisher Scientific, Inc., United States) coupled with an isotope ratio mass spectrometer (Finnigan Delta V Advantage, Thermo Fisher Scientific, Inc.). The total content of ^15^N in a specific organ type (leaf, stem, or root) was calculated as the product of DW and the ^15^N concentration of each organ type. Over the 20 days stress period, the ^15^N uptake flux was calculated based on the RGR, DW, and total contents of ^15^N in the root, stem, and leaf according to the equation of Liang et al. (2017). Partitioning among leaves, stems, and roots was examined relative to the whole-plant contents of ^15^N.

### Determination of the Activities of Enzymes Involved in N Assimilation

Enzyme activities during the process of N assimilation were determined spectrophotometrically. The activity of NR was monitored in leaves and roots according to the *in vitro* method described by Högberg et al. (1986). The activity of NiR was measured based on a previously described method (Ogawa et al., 1999). The GS activity was measured according to the method of Claussen and Lenz (1999), and GOGAT was assayed by the method described by Lin and Kao (1996).

### Quantitative Real-Time Polymerase Chain Reaction Analysis

Total RNA was extracted from leaf and root samples using an M5 Plant RNeasy Complex Mini Kit (Mei5 Biotechnology Co., Ltd., Beijing, China) according to the manufacturer’s instructions. We performed reverse transcription using the UEIrisIIRT-PCR System for the First-Strand cDNA Synthesis System (Suzhou US Everbright, Inc., Suzhou, China). The expression levels of genes involved in N metabolism and uptake were measured by quantitative real-time polymerase chain reaction (qRT-PCR) (Table 1). The qRT-PCR was performed on a LightCycler 96 Real-Time PCR System (Roche Diagnostics, Basel, Switzerland) using the AugeGreen™ qPCR Master Mix Kit (Suzhou US Everbright, Inc.) with β-actin as an internal standard. Three independent biological replicates were performed for each sample.

**TABLE 1:** Sequences of primers used in qRT-PCR.

Gene	Primer sequence (5′-3′)
*NRT1:1* F	CTGGCTGGTCCCACAGTTCTT
*NRT1:1* R	CTTCATTCCTTTCGGGCACTC
*NRT2*:*4* F	CAGAAGGTGAACCCGGAAG
*NRT2*:*4* R	CAAGTGGAACGTCCTCATGTG
*NRT2*:*5* F	TTGTGGTCCATCTAAGAACAAGGC
*NRT2*:*5* R	TCATCAGAGGGTCGGGTAACAG
*NRT2*:*7* F	TCTCCAGGCAGACGAGCATT
*NRT2:7* R	GGAGCAAGTGATACTGGTTTGTTTC
*NIR* F	GTCCATCCGCAGAAACAAGAAG
*NIR* R	GTTCCCCTGTGCCATACTCATC
*NR* F	CGATGACGACGAGAATGAGGAC
*NR* R	GCGGACCATAGACGAGTTACGAC
*GS* F	ATATCTGCTGGAGATGAACTGTGG
*GS* R	TGGACTTGGTGCTGTAGTTTGTG
*NADH*-*GOGAT* F	TGCCTAAGTTTATCAAGGTTATTCC
*NADH*-*GOGAT* R	CTCATCTTCCTCCTCGTGCTCT
*Fd*-*GOGAT* F	CGAAGGAAGAAGAAGACCACGC
*Fd*-*GOGAT* R	TTGCTGGTGCCTGTTGGGTT
β-*Actin* F	GGATTTGCTGGTGATGATGCT
β-*Actin* R	AGTTGCTCACTATGCCGTGCT

### Statistical Analysis

All data were analyzed using IBM SPSS Statistics 20 software (IBM Corp, Armonk, NY, United States) and then graphed with SigmaPlot 10.0 software for Windows (Systat Software, Inc., San Jose, CA, United States). The means of the treatments were compared by one-way analysis of variance (ANOVA), and differences between treatments were evaluated using Tukey’s multiple range test (*P* < 0.05).

## Results

### Effects of Exogenous Melatonin and Dopamine on Plant Growth

Under low-NO_3_^–^ stress, plant growth was severely inhibited in comparison with the normal control, leading to significant decreases in PL, TD, LN, TDW, and RGR of 33.4, 24.0, 33.2, 47.8, and 52.5%, respectively. However, exogenous melatonin and dopamine both significantly alleviated these declines compared with NO_3_^–^-deficient plants that had received no exogenous material. The values of PL, TD, LN, TDW, and RGR in the NO_3_^–^-deficient plants with 0.1 μmol/L exogenous melatonin were lower by 24.9, 14.2, 22.3, 30.3, and 29.4%, respectively, than those for the normal control. For NO_3_^–^-deficient plants with 100 μmol/L exogenous dopamine, these growth indices were lower by only 19.4, 6.0, 15.8, 4.4, and 3.4%, respectively. Therefore, both melatonin and dopamine significantly alleviated the inhibition attributable to low-NO_3_^–^ stress, although the effect of 100 μmol/L exogenous dopamine was more obvious. Moreover, under the normal NO_3_^–^ condition, melatonin and dopamine could also promote plant growth, but the effect was not as obvious as in the plants under the low NO_3_^–^ condition. In addition, due to the root foraging response, the RSR was increased significantly (by 46.3%) under NO_3_^–^ deficiency in comparison with the normal control, and this response was enhanced even more by the administration of exogenous dopamine (Table 2).

**TABLE 2:** Plant length (PL), trunk diameter (TD), leaf number (LN), total dry weight (TDW), root-to-shoot ratio (RSR), and relative growth rate (RGR) for plants grown 20 days under different N levels and material treatments.

Treatment	PL (cm)	TD (mm)	LN (No. plant^–1^)	TDW (g plant^–1^)	RSR (%)	RGR (g kg^–1^ d^–1^)
CK	30.83 ± 1.96*^a^*	2.33 ± 0.12*^b^*	18.4 ± 1.35*^a^*	2.28 ± 0.15*^b^*	20.22 ± 2.22*^c^*	62.90 ± 3.24*^bc^*
ST	20.52 ± 1.52*^c^*	1.77 ± 0.10*^d^*	12.3 ± 1.06*^c^*	1.19 ± 0.17*^d^*	29.58 ± 5.52*^ab^*	29.90 ± 7.30*^e^*
MCK	31.02 ± 1.73*^a^*	2.57 ± 0.14*^a^*	17.9 ± 1.52*^a^*	2.64 ± 0.24*^a^*	19.74 ± 1.43*^c^*	70.12 ± 4.54*^a^*
MST	23.14 ± 1.95*^b^*	2.00 ± 0.13*^c^*	14.3 ± 1.16*^b^*	1.59 ± 0.24*^c^*	29.18 ± 4.62*^b^*	44.41 ± 7.45*^d^*
DCK	31.14 ± 3.04*^a^*	2.64 ± 0.23*^a^*	17.6 ± 1.51*^a^*	2.55 ± 0.11*^a^*	18.95 ± 1.20*^c^*	68.52 ± 2.25*^ab^*
DST	24.84 ± 0.60*^b^*	2.19 ± 0.08*^bc^*	15.5 ± 0.71*^b^*	2.18 ± 0.14*^b^*	33.87 ± 2.38*^a^*	60.77 ± 3.31*^c^*

*Data are means ± SD (n = 10). Values not followed by same letter denote significant differences by Tukey’s multiple range tests (P < 0.05). CK, 2 mM K^15^NO_3_ solution; ST, 0.1 mM K^15^NO_3_ solution; MCK, 2 mM K^15^NO_3_ solution with 0.1 μmol/L melatonin; MST, 0.1 mM K^15^NO_3_ solution with 0.1 μmol/L melatonin; DCK, 2 mM K^15^NO_3_ solution with 100 μmol/L dopamine; DST, 0.1 mM K^15^NO_3_ solution with 100 μmol/L dopamine.*

### Effects of Exogenous Melatonin and Dopamine on Chlorophyll Content

**FIGURE 1:**
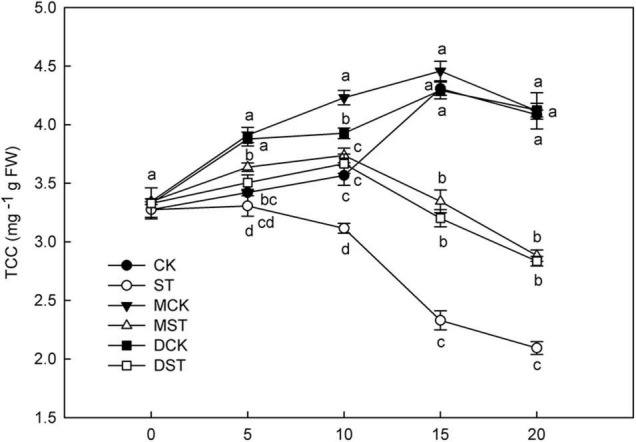
Effect of melatonin and dopamine on total chlorophyll concentration (TCC) under different N levels and material treatments. Data are means ± SD of five replicate samples. Values not followed by same letter denote significant differences by Tukey’s multiple range tests (*P* < 0.05). CK, 2 mM K^15^NO_3_ solution; ST, 0.1 mM K^15^NO_3_ solution; MCK, 2 mM K^15^NO_3_ solution with 0.1 μmol/L melatonin; MST, 0.1 mM/L K^15^NO_3_ solution with 0.1 μmol/L melatonin; DCK, 2 mM K^15^NO_3_ solution with 100 μmol/L dopamine; DST, 0.1 mM K^15^NO_3_ solution with 100 μmol/L dopamine.

N is a major element involved in the synthesis and metabolism of chlorophyll, and NO_3_^–^-deficiency triggers huge changes in total chlorophyll concentration. After 10 days of low NO_3_^–^ treatment, the chlorophyll content began to decrease significantly, and the application of exogenous melatonin or dopamine significantly alleviated the degradation of chlorophyll (Figure 1). At Day 20, the total chlorophyll concentration increased by 37.6 and 35.4% in plants treated with melatonin and dopamine under low-NO_3_^–^ stress, respectively.

### Effects of Exogenous Melatonin and Dopamine on Root Architecture

**FIGURE 2:**
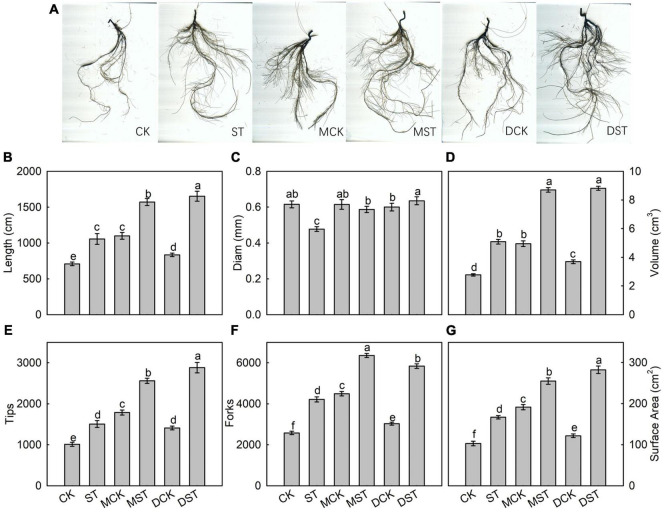
Effect of melatonin and dopamine on root system architecture of *Malus hupehensis* plants grown 20 days under different N levels and material treatments. **(A)** Root morphology. **(B)** Length. **(C)** Diam. **(D)** Volume. **(E)** Number of root tips. **(F)** Number of root forks. **(G)** Surface area. Data are means ± SD (*n* = 10). Values not followed by same letter denote significant differences by Tukey’s multiple range tests (*P* < 0.05). CK, 2 mM K^15^NO_3_ solution; ST, 0.1 mM K^15^NO_3_ solution; MCK, 2 mM K^15^NO_3_ solution with 0.1 μmol/L melatonin; MST, 0.1 mM K^15^NO_3_ solution with 0.1 μmol/L melatonin; DCK, 2 mM K^15^NO_3_ solution with 100 μmol/L dopamine; DST, 0.1 mM K^15^NO_3_ solution with 100 μmol/L dopamine.

After 20 days of exposure to the NO_3_^–^-deficient nutrient solution, the root system architecture had changed significantly (Figure 2A). Compared with the normal control, the ST, MST, and DST plants exhibited increases of 49.0, 121.8, and 133.1% in total root length; 83.4, 214.1, and 218.1% in root volume; 48.9, 153.3, and 185.0% in the number of root tips; 63.5, 146.8, and 126.6% in root forks; and 61.9, 147.5, and 174.1% in surface area (Figures 2B,D–G). However, the root diameter of the ST and MST plants decreased by 22.6 and 4.8%, respectively. In contrast, dopamine increased the root diameter by 3.2% (Figure 2C).

### Effects of Exogenous Melatonin and Dopamine on ^15^N Uptake and Distribution

**FIGURE 3:**
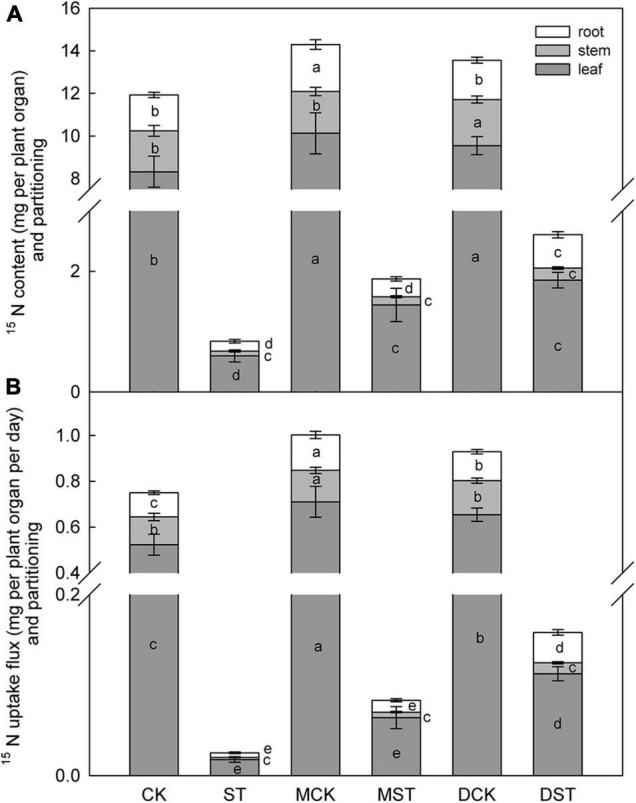
Effect of melatonin and dopamine on ^15^N content **(A)** and uptake flux **(B)** of *Malus hupehensis* plants grown 20 days under different N levels and material treatments. Data are means ± SD (*n* = 10). Values not followed by same letter denote significant differences by Tukey’s multiple range tests (*P* < 0.05). CK, 2 mM K^15^NO_3_ solution; ST, 0.1 mM K^15^NO_3_ solution; MCK, 2 mM K^15^NO_3_ solution with 0.1 μmol/L melatonin; MST, 0.1 mM K^15^NO_3_ solution with 0.1 μmol/L melatonin; DCK, 2 mM K^15^NO_3_ solution with 100 μmol/L dopamine; DST, 0.1 mM K^15^NO_3_ solution with 100 μmol/L dopamine.

The NO_3_^–^-deficient treatment significantly reduced ^15^NO_3_^–^ content in the whole plant (92.9%), and also reduced ^15^NO_3_^–^ uptake flux (96.6%) compared with samples collected under the normal condition. Under low-NO_3_^–^ stress, the accumulation of ^15^NO_3_^–^ was diminished in the leaf (92.8%), stem (90.1%), and root (95.9%). In addition, the proportion of ^15^NO_3_^–^ content in the stem compared to the whole plant decreased obviously under the low NO_3_^–^ condition. However, exogenous melatonin or dopamine was associated with obvious increases in the uptake and accumulation of ^15^NO_3_^–^ under the NO_3_^–^-deficient condition. When melatonin- and dopamine-treated plants were compared with no-material plants under the stress treatment, the ^15^NO_3_^–^ content were improved by 122.6 and 209.4%, and the ^15^NO_3_^–^ uptake flux increased by 230.2 and 528.2%, respectively. Furthermore, the accumulation and uptake of ^15^NO_3_^–^ increased in all parts of the plant (Figure 3). Under the low NO_3_^–^ condition, exogenous melatonin most obviously promoted ^15^NO_3_^–^ accumulation in leaves in comparison with the no-material plants. The ^15^NO_3_^–^ content and ^15^NO_3_^–^ uptake flux increased by 141.8 and 259.6%, respectively. Exogenous dopamine also significantly promoted ^15^NO_3_^–^ accumulation in leaves in comparison with the no-material plants. The ^15^NO_3_^–^ content and ^15^NO_3_^–^ uptake flux increased by 210.5 and 532.0%, respectively (Figure 3).

### Effects of Exogenous Melatonin and Dopamine on *N*-Metabolizing Enzyme Activities

**FIGURE 4:**
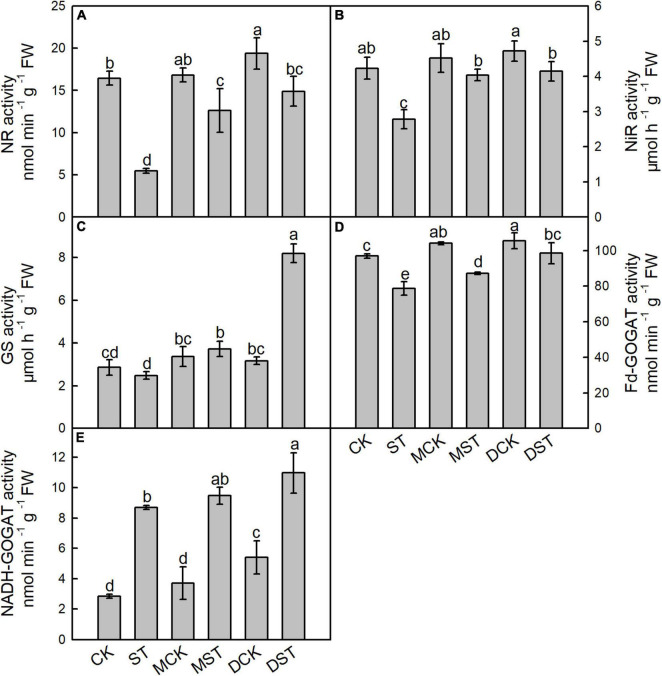
Effects of melatonin and dopamine on activities of nitrate reductase (NR; **A**), nitrite reductase (NiR; **B**), glutamine synthetase (GS; **C**), ferredoxin-dependent glutamate synthase (Fd-GOGAT; **D**), and nicotinamide adenine dinucleotide (NADH-GOGAT; **E**) in leaves under different treatment conditions. Data are means ± SD (*n* = 3). Values not followed by same letter denote significant differences by Tukey’s multiple range tests (*P* < 0.05). CK, 2 mM K^15^NO_3_ solution; ST, 0.1 mM K^15^NO_3_ solution; MCK, 2 mM K^15^NO_3_ solution with 0.1 μmol/L melatonin; MST, 0.1 mM K^15^NO_3_ solution with 0.1 μmol/L melatonin; DCK, 2 mM K^15^NO_3_ solution with 100 μmol/L dopamine; DST, 0.1 mM K^15^NO_3_ solution with 100 μmol/L dopamine.

**FIGURE 5:**
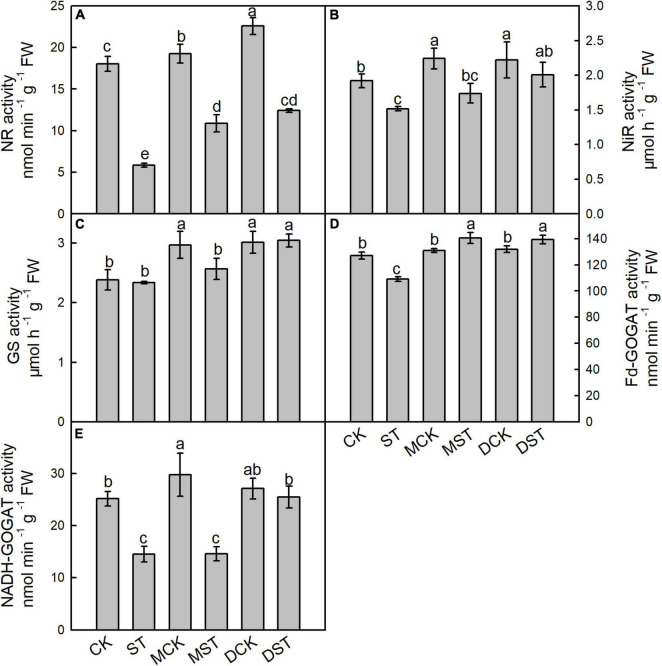
Effects of melatonin and dopamine on activities of nitrate reductase (NR; **A**), nitrite reductase (NiR; **B**), glutamine synthetase (GS; **C**), ferredoxin-dependent glutamate synthase (Fd-GOGAT; **D**), and nicotinamide adenine dinucleotide (NADH-GOGAT; **E**) in roots under different treatment conditions. Data are means ± SD (*n* = 3). Values not followed by same letter denote significant differences by Tukey’s multiple range tests (*P* < 0.05). CK, 2 mM K^15^NO_3_ solution; ST, 0.1 mM K^15^NO_3_ solution; MCK, 2 mM K^15^NO_3_ solution with 0.1 μmol/L melatonin; MST, 0.1 mM K^15^NO_3_ solution with 0.1 μmol/L melatonin; DCK, 2 mM K^15^NO_3_ solution with 100 μmol/L dopamine; DST, 0.1 mM K^15^NO_3_ solution with 100 μmol/L dopamine.

Enzymes such as NR, NiR, GS, Fd-GOGAT, and NADH-GOGAT play key roles in NO_3_^–^ reduction and N assimilation, and their enzyme activities changed dramatically under the NO_3_^–^-deficient condition. After 20 days of the NO_3_^–^-deficient treatment, the enzyme activity results indicated that low-NO_3_^–^ stress sharply reduced the activities of NR, NiR, and Fd-GAGOT in leaf and root. In the comparison between normal control and stressed plants, the activities of NR, NiR, GS, and Fd-GAGOT declined by 66.7, 34.3, 13.3, and 18.8% in leaf (Figures 4A–D) and 67.4, 20.9, 1.8, and 14.2% in root (Figures 5A–D), respectively. However, when melatonin or dopamine was applied to either the NO_3_^–^-normal or NO_3_^–^-deficient plants, the enzyme activity increased, especially in NO_3_^–^-deficient plants. By Day 20, the activities of NR, NiR, GS, and Fd-GOGAT in melatonin- and dopamine-treated leaves had increased by 130.1 and 171.7%, 45.2 and 48.9%, 50.2 and 230.7%, and 10.8 and 25.3% (Figures 4A–D), respectively, in comparison with the corresponding no-material plants under low-NO_3_^–^ stress. In root, the corresponding values were 85.7 and 111.7%, 14.5 and 32.2%, 9.7 and 30.2%, and 28.9 and 27.9%, respectively (Figures 5A–D). In addition, the activity of NADH-GOGAT declined by 42.2% in root (Figure 5E) and increased by 206.8% in leaf (Figure 4E); however, Fd-GOGAT is the enzyme that plays the major role in leaves. All of these results demonstrated that low-NO_3_^–^ stress slowed the processes of NO_3_^–^ reduction and N assimilation in leaves. However, supplementation with melatonin or dopamine substantially alleviated these responses, and dopamine was more effective than melatonin.

### Effects of Melatonin and Dopamine on Transcriptional Regulation of Genes Involved in N Metabolism and Transport

**FIGURE 6:**
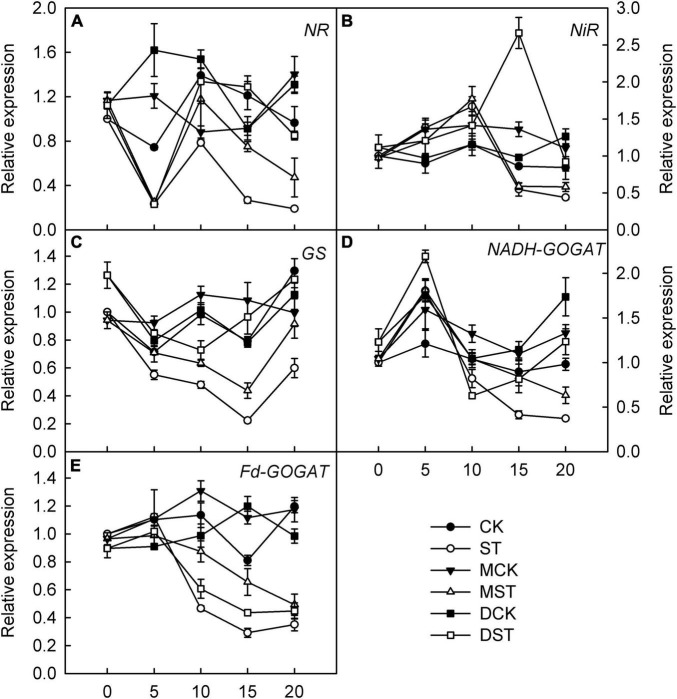
Effects of melatonin and dopamine on expression of key genes involved in N-assimilation in leaves under different treatment conditions. **(A)** NR. **(B)** NiR. **(C)** GS. **(D)** NADH-GOGAT. **(E)** Fd-GOGAT. Total RNA was isolated from samples at specified time points (0–20 days), converted to cDNA, and subjected to qRT-PCR. Data are means ± SD (*n* = 3). CK, 2 mM K^15^NO_3_ solution; ST, 0.1 mM K^15^NO_3_ solution; MCK, 2 mM K^15^NO_3_ solution with 0.1 μmol/L melatonin; MST, 0.1 mM K^15^NO_3_ solution with 0.1 μmol/L melatonin; DCK, 2 mM K^15^NO_3_ solution with 100 μmol/L dopamine; DST, 0.1 mM K^15^NO_3_ solution with 100 μmol/L dopamine.

**FIGURE 7:**
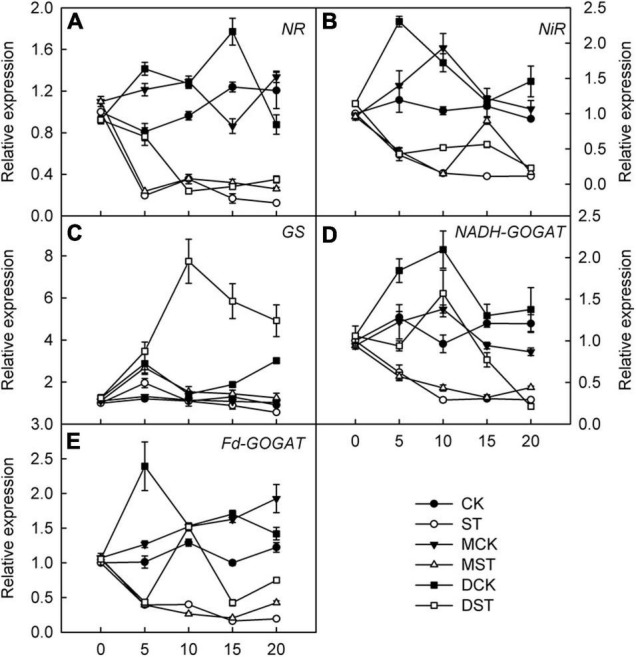
Effects of melatonin and dopamine on expression of key genes involved in N-assimilation in roots under different treatment conditions. **(A)** NR. **(B)** NiR. **(C)** GS. **(D)** NADH-GOGAT. **(E)** Fd-GOGAT. Total RNA was isolated from samples at specified time points (0–20 d), converted to cDNA, and subjected to qRT-PCR. Data are means ± SD (*n* = 3). CK, 2 mM K^15^NO_3_ solution; ST, 0.1 mM K^15^NO_3_ solution; MCK, 2 mM K^15^NO_3_ solution with 0.1 μmol/L melatonin; MST, 0.1 mM K^15^NO_3_ solution with 0.1 μmol/L melatonin; DCK, 2 mM K^15^NO_3_ solution with 100 μmol/L dopamine; DST, 0.1 mM K^15^NO_3_ solution with 100 μmol/L dopamine.

Furthermore, we studied the internal molecular responses induced by low-NO_3_^–^ stress, as demonstrated by the changing transcriptional regulation patterns of key genes implicated in N uptake and metabolism. After 5–10 days of exposure to the NO_3_^–^-deficient nutrient solution, we found that the transcript levels of all investigated genes related to N metabolism (*NR*, *NiR*, *GS*, *NADH-GOGAT*, and *Fd-GAGOT*) in the leaves and roots of the no-material plants were significantly lower than those of the normal control plants (Figures 6A–E, 7A–E). However, their relative expression was higher in the melatonin- and dopamine-treated plants than in the no-material plants, indicating that melatonin and dopamine promoted the expression of genes associated with N metabolism under the low NO_3_^–^ condition (Figures 6A–E, 7A–E). In addition, we found that dopamine could especially increase the transcript levels of *GS* after 5 days of treatment (Figures 6C, 7C).

**FIGURE 8:**
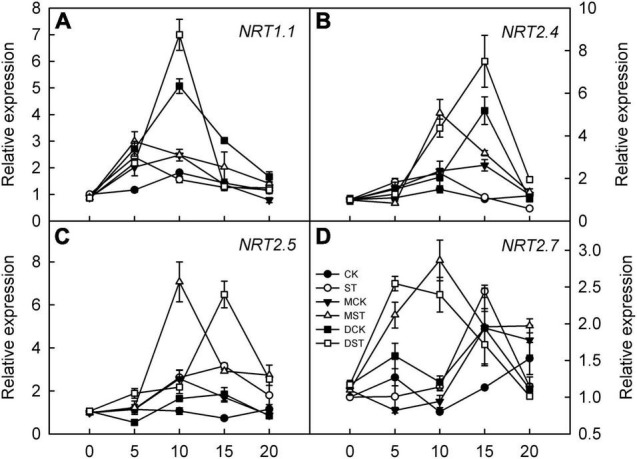
Effects of melatonin and dopamine on expression of key genes involved in N-uptake in leaves under different treatment conditions. **(A)** NRT1.1. **(B)** NRT2.4. **(C)** NRT2.5. **(D)** NRT2.7. Total RNA was isolated from samples at specified time points (0–20 days), converted to cDNA, and subjected to qRT-PCR. Data are means ± SD (*n* = 3). CK, 2 mM K^15^NO_3_ solution; ST, 0.1 mM K^15^NO_3_ solution; MCK, 2 mM K^15^NO_3_ solution with 0.1 μmol/L melatonin; MST, 0.1 mM K^15^NO_3_ solution with 0.1 μmol/L melatonin; DCK, 2 mM K^15^NO_3_ solution with 100 μmol/L dopamine; DST, 0.1 mM K^15^NO_3_ solution with 100 μmol/L dopamine.

**FIGURE 9:**
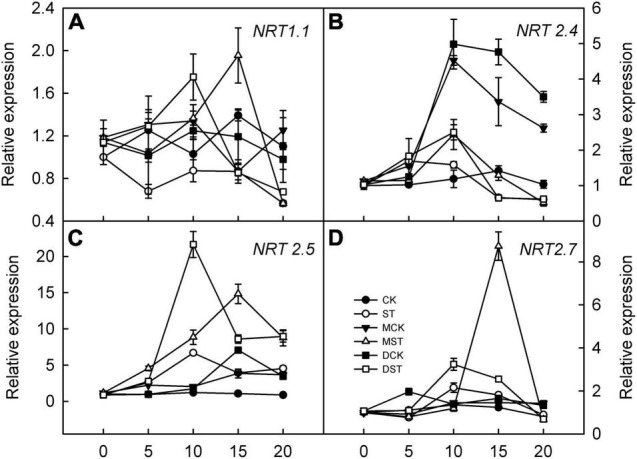
Effects of melatonin and dopamine on expression of key genes involved in N-uptake in roots under different treatment conditions. **(A)** NRT1.1. **(B)** NRT2.4. **(C)** NRT2.5. **(D)** NRT2.7. Total RNA was isolated from samples at specified time points (0–20 days), converted to cDNA, and subjected to qRT-PCR. Data are means ± SD (*n* = 3). CK, 2 mM K^15^NO_3_ solution; ST, 0.1 mM K^15^NO_3_ solution; MCK, 2 mM K^15^NO_3_ solution with 0.1 μmol/L melatonin; MST, 0.1 mM K^15^NO_3_ solution with 0.1 μmol/L melatonin; DCK, 2 mM K^15^NO_3_ solution with 100 μmol/L dopamine; DST, 0.1 mM K^15^NO_3_ solution with 100 μmol/L dopamine.

The expression of genes involved in regulating NO_3_^–^ transport, that is, *NRT2.4*, *NRT2.5*, and *NRT2.7*, was significantly upregulated in response to NO_3_^–^ deficiency in both leaves and roots; however, exogenous melatonin and dopamine amplified this enhancement effect and the relative expression levels were higher. Transcript levels peaked between days 10 and 15 of treatment, and the relative expression levels then gradually declined (Figures 8B–D, 9B–D). In addition, the relative expression level of N transporter *NRT1.1* was significantly upregulated in leaves, and exogenous melatonin and dopamine further promoted its upregulation under low-NO_3_^–^ stress (Figure 8A), except in roots (Figure 9A).

## Discussion

N availability is usually an important limiting factor of plant growth, unless the root system forms a symbiotic relationship with N-fixing microorganisms. However, only a very small proportion (0.00024%) of Earth’s N is available for plants in the soil environment, as well as for microorganisms and animals (Viktor and Cramer, 2005). More than half of the world’s population relies on crops grown with synthetic N fertilizers. Over the past century, reliable supplies of N and other nutrients necessary for plant growth have allowed farmers to dramatically increase crop yields per unit of land, boosting economic development and allowing populations to increase (Zhang et al., 2015). Unfortunately, crops can only use 30–50% of the N fertilizer applied to soil (Miller and Cramer, 2005). Thus, excessive use of N fertilizer increases planting costs, results in serious environmental pollution, and endangers crop growth and human health (Galloway et al., 2008). Increasing NUE is one of the most effective ways to increase crop productivity while reducing environmental degradation. However, such improvements are conditional not only on management innovations, but also on genetic breeding and understanding the important role of hormones and other substances in the plant N cycle. Therefore, we examined the effects of melatonin and dopamine on the growth, photosynthesis, root system structure, and N metabolism of NO_3_^–^-deficient *M. hupehensis* seedlings.

Growth inhibition is an important indicator of environmental stress in plants. Li et al. (2016) reported that 0.1 μmol/L melatonin promoted plant growth under K-deficiency stress. Additionally, the application of 100 μmol/L exogenous dopamine reduced the inhibitory effect of N deficiency on plant growth significantly (Liu X. M. et al., 2020). However, this is the first systematic study on how melatonin and dopamine ameliorate the effects of low-NO_3_^–^ stress in *M. hupehensis*. We found that the growth indicators were significantly lower in NO_3_^–^-deficient plants than in plants grown under normal conditions. This result was similar to the observations of previous studies (e.g., Liu X. M. et al., 2020). However, the application of exogenous melatonin and dopamine markedly mitigated these reductions, and dopamine was the more effective of the two. In addition, NO_3_^–^ deficiency can promote root growth and thus increase the absorption area. This phenomenon is called the root foraging response (Giehl et al., 2014). We found that the RSR increased significantly (by 46.3%) in comparison with the normal control under NO_3_^–^ deficiency, and that this response was enhanced by the administration of exogenous dopamine.

Chlorophyll is the main photosynthetic pigment in green plants and algae, but is easily destroyed during photosynthesis and under environmental stress. Therefore, it is essential to maintain functional chlorophyll for the growth and development of plants. N is an important component of chlorophyll, and a lack of N hinders chlorophyll synthesis, resulting in the yellowing of leaves and weakening (or even cessation) of photosynthesis (Liu X. M. et al., 2020). In this study, exogenous melatonin and dopamine can protect plants from the damage caused by low-NO_3_^–^ stress, by maintaining high concentrations of photosynthetic pigment. Furthermore, recent research has shown that this result can be attributed to downregulation by melatonin of the genes involved in chlorophyll metabolism (*CHLASE, PPH, PAO*, and *Chl-PRX*) (Arnao and Hernandez-Ruiz, 2014; Sharma et al., 2020), and to the fact that melatonin also increases the biosynthesis of carotenoids by upregulating genes in the carotenoid biosynthetic pathway, phytoene synthase 1 (*PSY1*) and carotenoid isomerase (*CRTISO*) (Sun et al., 2015). In addition, exogenous dopamine had strong inhibitory effects on the upregulation of the chlorophyll degradation gene (Shiva et al., 2016) and senescence-associated gene under drought conditions (Liang et al., 2018a). All of these results verify the protective effects of melatonin and dopamine on plant photosynthesis under stress conditions at the molecular level.

Root developmental plasticity is essential for optimizing NO_3_^–^ capture in continuously changing and NO_3_^–^-deficient soil environments (Gifford et al., 2013). Plant roots preferentially colonize N-rich regions through directed lateral root development in growth substrates with uneven N availability. Several studies have found that NO_3_^–^ mainly stimulates lateral root elongation and that NH_4_^+^ induces lateral root branching, supporting the view that the root configurations promoted by the two main inorganic forms of N are mutually complementary (Zhang and Forde, 1998; Ying and Nicolaus, 2017). Under nutrient deficiency, plant roots adapt by constantly adjusting their physiological and structural characteristics, and the degree of adaptation depends on their ability to change their configuration (Jia et al., 2019). Moreover, several root architecture responses are related to changes in phytohormone flux or balance. In particular, adaptive responses of roots to different nutrient supplies can be induced by changing the auxin transport or signal transduction (Giehl et al., 2012). However, melatonin and auxin are synthesized with tryptophan as a precursor, and studies have shown that both melatonin and dopamine regulate the expression of various factors (enzymes, receptors, and transcription factors) participating in phytohormone biosynthesis and catabolism, such as of auxins (Wang et al., 2016), gibberellins (Zhang et al., 2014), and abscisic acid and ethylene (Liu X. M. et al., 2020). Therefore, the application of exogenous melatonin or dopamine significantly promotes root system development and favors N absorption by plants under the NO_3_^–^-deficient condition. Under low nitrate conditions, the root system architecture had changed significantly. However, the total root length, root volume, the numbers of root tips, and surface area were higher in the dopamine treated plants than in the melatonin treated plants. This may lead to greater effectiveness of dopamine under nitrate stress in comparison to melatonin.

Various studies have suggested that nutrient uptake and transport by plants are generally inhibited by various abiotic stresses (Sánchez-Rodríguez et al., 2010; Kanwar et al., 2018; Qi et al., 2019). Exogenous melatonin has been shown to enable stressed plants to maintain significantly higher K levels in leaves in comparison to plants not exposed to melatonin under K- and all nutrient-deficient conditions (Li et al., 2016). Furthermore, excess NO_3_^–^ has been shown to reduce levels of phosphorus and magnesium, although this is usually mitigated by exogenous melatonin (Zhang et al., 2017). Similarly, exogenous dopamine can alleviate the inhibitory effects of nutrient deficiency on the absorption and accumulation of large and trace elements under salt stress (Li et al., 2015), and also prominently increases the concentrations, uptake, and transport of nutrients, while simultaneously altering their distribution within the whole plant, under drought conditions (Liang et al., 2018a). Through an investigation of stable isotopes, Liang et al. (2018b) confirmed that the concentration, uptake activity, and utilization rate of δ^15^N were obviously increased by the addition of exogenous melatonin under drought stress. We also investigated stable isotopes to determine the ^15^NO_3_^–^ content: the ^15^NO_3_^–^ uptake flux was obviously increased by the addition of exogenous melatonin or dopamine under the low NO_3_^–^ condition. The NO_3_^–^-deficient treatment significantly reduced the concentration of ^15^NO_3_^–^ in the whole plant after 20 days, and also reduced ^15^NO_3_^–^ uptake flux in comparison with plants under the normal condition. In addition, the proportion of ^15^NO_3_^–^ in the stem relative to that in the whole plant decreased obviously under the low NO_3_^–^ condition. The accumulation of N in roots and leaves is beneficial for the maintenance of normal plant growth. Exogenous melatonin and dopamine both distinctly increased the uptake and accumulation of ^15^NO_3_^–^ under NO_3_^–^ deficiency. Moreover, the accumulation and uptake of ^15^NO_3_^–^ increased in all parts of the plant, and exogenous melatonin very obviously promoted ^15^NO_3_^–^ accumulation in leaves in comparison with the no-material plants under the low-NO_3_^–^ stress condition. However, exogenous dopamine was more effective at promoting N accumulation, and the effect was most striking in roots. This phenomenon is well explained by the only slight increase in root diameter, and by the fact that the RSR was enhanced even more significantly by the administration of exogenous dopamine under the NO_3_^–^-deficient condition. Therefore, melatonin and dopamine not only promoted ^15^NO_3_^–^ absorption in the whole plant, but also altered the ^15^NO_3_^–^ distribution among the plant groups.

For many plants, after NO_3_^–^ is absorbed into the root, NO_3_^–^ accumulation in the root is limited; most of it is transported to the aboveground parts, where the first step is reduction of NO_3_^–^ to NO_2_^–^ by NO_3_^–^ reductase in the cytoplasm, followed by reduction to NH_4_^+^ via NO_3_^–^ reductase in the plastids (Wang et al., 2008). The NH_4_^+^ reduced from NO_3_^–^ is assimilated into amino acids by the GS/GOGAT cycle, which is a crucial step for converting inorganic N into organic N in plants, and the predominant GS/GOGAT isoenzymes are chloroplastic GS2/Fd-GOGAT and cytosolic GS1/NADH-GOGAT (Xu et al., 2012). Liu X. M. et al. (2020) confirmed that the activities of NR, NiR, GS, and GOGAT were decreased to varying degrees under NO_3_^–^ deficiency stress in *M. hupehensis* plants. Similarly, our data showed that the activities of key enzymes involved in N assimilation were decreased under the NO_3_^–^-deficient conditions. However, when exogenous melatonin or dopamine was applied to either NO_3_^–^-normal or NO_3_^–^-deficient plants, enzyme activity increased, in comparison to the untreated plants. And the activities of key enzymes involved in N assimilation were higher in the dopamine treated plants than in the melatonin treated plants, both in leaf and root. Our results demonstrate that melatonin and dopamine improve the uptake of NO_3_^–^ in *M. hupehensis* plants by enhancing the activities of key enzymes involved in N assimilation, so that plants can absorb and use N better under NO_3_^–^-deficient conditions, and the effect of dopamine may be better.

In addition, we studied the internal molecular responses induced by low-NO_3_^–^ stress, including the transcript levels of genes related to N metabolism and uptake in plant leaf and root. Melatonin has been shown to promote the expression of genes involved in N metabolism, thus relieving the inhibition induced by drought stress (Liang et al., 2018b). Similarly, we found that both melatonin and dopamine enhanced the expression of *NR*, *NiR*, *GS*, *Fd-GOGAT*, and *NADH-GOGAT* under low-NO_3_^–^ stress. Previous research identified 53 *NRT1* genes and 7 *NRT2* genes in the *Arabidopsis thaliana* genome. Most NRT1 transporters display low affinity for NO_3_^–^, although NRT1.1/NPF6.3/CHLORATE RESISTANT 1 (CHL1), the first NO_3_^–^ transporter discovered in plants, exhibits a characteristic dual affinity (Tsay et al., 1993; Su et al., 2020). *AtNRT1.1* has the capacity to transport multiple substrates. It not only uptakes NO_3_^–^ for plant growth, but also reveals auxin transport activity and participates in NO_3_^–^-modulated root development (Krouk et al., 2010; Zhang et al., 2019). However, we did not observe significant upregulation at the transcription level of *NRT1.1* in roots, and neither melatonin nor dopamine increased the transcription level of *NRT1.1* significantly. In addition, *AtNRT1.1* is also strongly expressed in guard cells, which regulate stomatal opening and thus affect transpiration (Guo et al., 2003). In leaves, drought stress has been shown to inhibit the transcript levels of *NRT1.1*, and exogenous melatonin has been shown to promote the expression of genes associated with *NRT1.1* (Liang et al., 2018b). We found that *NRT1.1* transcription was significantly upregulated, and that exogenous melatonin and dopamine enhanced the transcription of *NRT1.1* in leaves, which could contribute to the maintenance of photosynthesis in NO_3_^–^-deficient plants.

In general, NRT2s exhibit high-affinity NO_3_^–^ transport activity when expressed in oocytes; however, some may exhibit only low-affinity NO_3_^–^ transport activity (Fu et al., 2015). *ATNRT2.1* is a high-affinity NO_3_^–^ uptake gene in *A. thaliana* (Cerezo et al., 2001). Moreover, *AtNRT2.4* and *AtNRT2.5* also participate in high-affinity NO_3_^–^ uptake (Kiba et al., 2012). *AtNRT2.5* expression is strongly induced, and it becomes the primary transporter for high-affinity uptake after a long period of NO_3_^–^ deficiency (Lezhneva et al., 2014). After 5 days of low NO_3_^–^ treatment, we discovered that the expression levels of *NRT2.4* and *NRT2.5* first increased, and then decreased, in both leaf and root. The peaks occurred on day 10 or 15. However, exogenous melatonin and dopamine strongly upregulated *NRT2.4* and *NRT2.5* leaf and root. In addition, *NRT2.7* is a seed-specific, high-affinity NO_3_^–^ transporter that controls NO_3_^–^ content in mature seeds of *A. thaliana*, and has been shown to be related to the accumulation and oxidation of proanthocyanidins (Liang et al., 2015). Some studies have found that melatonin can upregulate the expression of *NRT2.7* in plants under drought stress (Huang et al., 2018; Liang et al., 2018b). Our results revealed that exogenous melatonin and dopamine can significantly upregulate the transcription levels of *NRT2.7* in *M. hupehensis*. The aforementioned results indicate that both melatonin and dopamine regulate the expression of genes involved in N-metabolism and uptake in *M. hupehensis* seedlings under the low NO_3_^–^ condition, thereby maintaining their growth and development.

## Conclusion

This study confirmed that melatonin and dopamine can both alleviate the inhibition of plant growth and development, chlorophyll degradation, and decreased N uptake caused by low-NO_3_^–^ stress. Moreover, these substances both have the ability to significantly change root architecture and effectively upregulate the expression levels of genes involved in N-metabolism and transport in comparison with plants without any allogenic material under low-NO_3_^–^ stress. We systematically investigated the ability of dopamine and melatonin, as potential plant hormones, to increase NUE in *M. hupehensis* under low-NO_3_^–^ stress. Further exploration of the roles of melatonin and dopamine in nutrient absorption may improve agriculture in the future.

## Data Availability Statement

The original contributions presented in the study are included in the article/supplementary material, further inquiries can be directed to the corresponding author/s.

## Author Contributions

BL conceived and designed the experiments, provided financial support, and helped perform the analysis with constructive discussions. PD performed the experiments with assistance from BY, YC, RH, JJ, and XH. PD analyzed the data and wrote the manuscript. JX provided materials and laboratory apparatus. All the authors contributed to the article and approved the submitted version.

## Conflict of Interest

The authors declare that the research was conducted in the absence of any commercial or financial relationships that could be construed as a potential conflict of interest.

## Publisher’s Note

All claims expressed in this article are solely those of the authors and do not necessarily represent those of their affiliated organizations, or those of the publisher, the editors and the reviewers. Any product that may be evaluated in this article, or claim that may be made by its manufacturer, is not guaranteed or endorsed by the publisher.
